# Autophagy enhances mesenchymal stem cell-mediated CD4^+^ T cell migration and differentiation through CXCL8 and TGF-β1

**DOI:** 10.1186/s13287-019-1380-0

**Published:** 2019-08-23

**Authors:** Shuizhong Cen, Peng Wang, Zhongyu Xie, Rui Yang, Jinteng Li, Zhenhua Liu, Shan Wang, Xiaohua Wu, Wenjie Liu, Ming Li, Su’an Tang, Huiyong Shen, Yanfeng Wu

**Affiliations:** 1 Department of Orthopedics, Sun Yat-sen Memorial Hospital, Sun Yat-sen University, 107# Yan Jiang Road West, Guangzhou, 510120 People’s Republic of China; 2 Center for Biotherapy, Sun Yat-sen Memorial Hospital, Sun Yat-sen University, 107# Yan Jiang Road West, Guangzhou, 510120 People’s Republic of China; 3 Department of Orthopedics, The Eighth Affiliated Hospital, Sun Yat-sen University, 3025# Shen Nan Road, Shenzhen, 518033 People’s Republic of China; 4 Department of Orthopedics, ZhuJiang Hospital of Southern Medical University, 253# Industry Avenue, Guangzhou, 510282 People’s Republic of China

**Keywords:** Autophagy, Mesenchymal stem cells, Immunomodulatory, Migration, CD4^+^ T cells

## Abstract

**Background:**

Mesenchymal stem cells (MSCs) have been recognized as a promising tool for the treatment of various inflammatory disorders and autoimmune diseases. Stress conditions affect immune-mediated treatment and activate autophagy in MSCs. However, whether autophagy affects the MSC-mediated recruitment and differentiation of CD4^+^ T cells remains elusive.

**Methods:**

MSCs were pretreated with 3-methyladenine (3-MA) and rapamycin to regulate autophagy, and then co-cultured with CD4^+^ T cells. CD4^+^ T cell migration and differentiation were detected by flow cytometry. Further, gene expression levels of well-known chemokines were analyzed by quantitative real-time PCR. Enzyme-linked immunosorbent assays and western blot analysis were performed to detect C-X-C motif chemokine ligand 8 (CXCL8) and transforming growth factor (TGF)-β1 protein levels. An exogenous antibody and short hairpin RNA were used to regulate CXCL8 and TGF-β1 levels, which enabled us to evaluate how autophagy affected MSC-mediated CD4^+^ T cell migration and differentiation.

**Results:**

3-MA inhibited autophagy in MSCs, which was activated by rapamycin. Rapamycin increased the migration of CD4^+^ T cells, whereas 3-MA decreased their migration. Mechanistically, we found that autophagy strengthened CXCL8 secretion, and the addition of exogenous CXCL8 and an anti-CXCL8 antibody eliminated the difference of CD4^+^ T cell migration among groups. Further, the ratio of regulatory T (Treg) cells was increased in rapamycin-pretreated MSCs, but the ratio of T helper 1 (Th1) cells was decreased, while pretreatment of MSCs with 3-MA induced the opposite effect compared with the control group. TGF-β1 overexpression and knockdown using lentiviruses rectified the differences in the ratios of Treg and Th1 cells among the groups.

**Conclusion:**

This study demonstrates that autophagy of mesenchymal stem cells mediates CD4^+^ T cell migration and differentiation through CXCL8 and TGF-β1, respectively. These results provide a potential new strategy for improving MSC-mediated therapy.

**Electronic supplementary material:**

The online version of this article (10.1186/s13287-019-1380-0) contains supplementary material, which is available to authorized users.

## Background

Mesenchymal stem cells (MSCs) are recognized as pluripotent progenitor cells that can differentiate into osteoblasts, chondrocytes, and adipocytes [1]. Due to their immunoregulatory function and limited immunogenicity, MSC-based therapies have been applied successfully in many inflammatory and autoimmune diseases [2, 3]. Despite the clinical potential of MSCs, their clinical efficacy remains limited due to the effects of the in vivo microenvironment. Significant attention has been paid to exploring the mechanism of MSC-based therapy. In addition, some studies have indicated that the stress microenvironment can also influence autophagy [4]. However, whether autophagy affects MSC-based therapies must be further explored.

Autophagy is a basic cellular homeostatic process involved in different physiological and pathological conditions [5]. When cells encounter stressful conditions, such as inflammation, starvation, or growth factor deprivation, autophagy is induced to recycle proteins and to allow dysfunctional organelles to adapt to the conditions. However, some studies hold that autophagy also has negative effects on various aspects of cell physiology [6]. An increasing body of evidence has demonstrated that autophagy plays important roles in both innate and adaptive immunity involving numerous leukocyte populations including T cells, B cells, and natural killer cells [7, 8]. Moreover, autophagy reportedly affects the MSC apoptosis and differentiation and is regarded as a protective mechanism under physiological conditions. We previously reported that autophagy in MSCs improved the immunosuppression of CD4^+^ T cells [9], but whether autophagy affects other aspects of MSC-mediated immunomodulation remains unknown and requires further study.

An important aspect of MSC-mediated immunomodulation is the recruitment and polarization of CD4^+^ T cells [10]. Chemokines are a family of small peptides that can regulate chemotaxis and other cellular functions [11]. C-X-C motif chemokine ligand 8 (CXCL8, also known as interleukin [IL]-8), the first chemokine to be identified, was initially demonstrated to play an important role in neutrophil activation and migration [12]. Further studies revealed that CXCL8 could also recruit T leukomonocytes, monocytes, etc. [13]. In addition to chemokines, MSCs also secrete enormous amounts of immune regulatory cytokines such as transforming growth factor (TGF)-β1, IL-12, and prostaglandin E2, which play key roles in MSC-mediated immunomodulation. In addition, most studies have focused primarily on CD4^+^ T cells, as they are critical effectors of the immune adaptive response [14]. Naïve CD4^+^ T cells can proliferate and differentiate into various T cell lineages, including T helper 1 (Th1), Th2, Th17, and regulatory T (Treg) cells, which exert different effects in immunity [15]. Most studies have confirmed that Th1 and Th17 cells are proinflammatory, while Th2 and Treg cells are anti-inflammatory in the host. An imbalance of Th1/Th2/Th17/Treg cells could cause an abnormal immune response and might be involved in some diseases such as ankylosing spondylitis [16].

In this study, we demonstrated that autophagy in MSC can regulate the recruitment and polarization of CD4^+^ T cells. We found that the inhibition of autophagy using 3-methyladenine (3-MA) weakened MSC-mediated CD4^+^ T cell recruitment, while the induction of autophagy using rapamycin induced the opposite effect and was mediated by CXCL8. Moreover, the 3-MA treatment induced a lower ratio of Treg cells and a higher ratio of Th1 cells, whereas the treatment with rapamycin resulted in a higher ratio of Treg cells and a lower ratio of Th1 cells compared with the control group due to the secretion of TGF-β1. Consistent with these results, activating autophagy in MSCs suppressed the concentration of inflammatory cytokines in a co-culture system. Thus, we speculate that autophagy is a critical regulator of MSC-mediated immunomodulation, which may be a potential new strategy for improving MSC-mediated therapy.

## Materials and methods

### Cell isolation and culture (MSCs and peripheral blood mononuclear cells)

This study was approved by the ethics committee of Sun Yat-Sen Memorial Hospital (Sun Yat-Sen University, Guangzhou, China). Eighteen healthy donors (10 men, 8 women) between the ages of 20 and 30 years were recruited for the study and signed the formed consent after being informed of the possible risks and study objectives. Bone marrow was extracted under sterile conditions, and MSCs were isolated and purified from the bone marrow samples using density-gradient centrifugation, as reported previously [9]. The MSCs were expanded, and cells from passages 3–5 were used for all subsequent experiments. Peripheral blood mononuclear cells (PBMCs) were isolated from the peripheral blood samples of another 18 healthy donors using Ficoll-Hypaque gradient centrifugation. After determining the cell numbers, the cell pellet was mixed in 80 μL of buffer and 20 μL of CD4 microbeads per 10^7^ total cells. The cell suspensions were mixed thoroughly and incubated at 4 °C for 15 min. Then, the cells were washed with buffer, and resuspended in 500 μL of buffer after centrifugation at 300×*g* for 10 min. The cell suspension was applied onto the pre-wet column placed in the MACS separator, and the column was washed with 500 μL of buffer three times. The column was removed from the separator, and the labeled cells were flushed out into a new tube with 1 mL of buffer. The labeled cells were collected for the following experiments, and their purity was assessed by flow cytometry.

### Cell culture

MSCs were seeded in 12-well plates and exposed to different treatments for 24 h. After being washed thoroughly with phosphate-buffered saline (PBS), MSCs were co-cultured with purified CD4^+^ T cells at a ratio of 1:10 MSCs (0.5 × 10^5^cells): CD4^+^ T cells (5 × 10^5^cells) in 2 mL RPMI-1640 medium. It is worth mentioning that all the co-culture experiments were performed in an allogeneic manner. To stimulate T cell proliferation, purified anti-CD3 (0.2 μg/mL) and anti-CD28 (1 μg/mL, BD Pharmingen) antibodies and recombinant human IL-2 (500 IU/ml) were added to the co-culture system. On the fifth day of co-culture, the cells were collected to analyze the ratio of Treg, Th1, Th2, and Th17 cells. In addition, the culture supernatant was collected for cytokine measurements using a Cytometric Bead Array (CBA) Kit (BD Biosciences), as described below.

### Flow cytometry

Collected MSCs were incubated for 30 min at room temperature with the following specific antibodies: PE mouse anti-human CD29, FITC rat anti-human CD44, FITC mouse anti-human CD105, FITC rat anti-human CD45, APC mouse anti-human CD34, and PE mouse anti-human HLA-DR (all from BD Pharmingen). As a control, the cells were stained with the appropriate isotype antibodies. At the end of co-culture, the CD4^+^ T cell apoptosis was analyzed by using an Annexin V-PE Apoptosis Detection Kit I (BD Biosciences) according to the manufacturer’s instructions. To detect Treg cells, a Human Regulatory T Cell Staining Kit (eBioscience) containing an anti-CD4-FITC/CD25-APC cocktail and anti-Foxp3-PE was used according to the manufacturer’s instruction. In addition, we used a Human Th1/Th2/Th17 Phenotyping kit (BD Pharmingen) to analyze the T helper cell subsets. All samples were analyzed using a BD Biosciences Influx cell sorter.

### Tri-lineage differentiation potential of MSCs

#### Osteogenic differentiation

MSCs were seeded on 12-well plates at a concentration of 1.5 × 10^4^ cells/cm^2^ and induced in osteogenic differentiation medium consisting of DMEM with 10% FBS, 100 IU/mL penicillin, 100 IU/mL streptomycin, 0.1 μM dexamethasone, 10 mM β-glycerol phosphate, and 50 μM ascorbic acid (Sigma-Aldrich). The medium was replaced every 3 days, and Alizarin Red S staining was used to detect de novo bone matrix formation on day 21.

#### Chondrogenic differentiation

MSCs were seeded as high-density pellets (5 × 10^5^ cells) in serum-free chondrogenic medium consisting of high-glucose DMEM with 1% ITS-Premix (Corning), 50 mg/L ascorbic acid (Sigma), 1 mM sodium pyruvate (Sigma), 100 nM dexamethasone (Sigma), and 10 ng/mL recombinant human transforming growth factor (TGF)-β3 (R&D) for 21 days. Toluidine blue staining was used to confirm the chondrogenic differentiation.

#### Adipogenic differentiation

MSCs were induced in specific medium containing DMEM supplemented with 10% FBS, 1 μM dexamethasone (Sigma), 10 μg/ml insulin (Sigma), 0.5 mM 3-isobutyl-1-methylxanthine (Sigma), and 0.2 mM indomethacin (Sigma). Oil Red O staining (ORO) was used to detect the intracellular fat droplets on day 21.

### 3-MA and rapamycin preparation and MSC pretreatment

To regulate autophagy in MSC, 3-methyladenine (3-MA) and rapamycin were used in our experiments. 3-MA (10 mM) was dissolved in the culture medium, and rapamycin (3 μM) was dissolved in dimethyl sulfoxide (DMSO). Besides, the medium containing only 0.1% DMSO was defined as the DMSO group (Additional file 5). MSCs cultured in medium without these agents were used as a control group.

### Cell proliferation assay

MSCs were digested and seeded in 96-well plates and treated with different drugs for the indicated times. Cell proliferation was detected by using a Cell Counting Kit-8 (CCK-8) assay (Dojindo Molecular Technologies) according to the manufacturer’s instructions. Medium without cells was used as a negative control.

### Formation and quantification of exogenous GFP-LC3 vacuoles

MSCs were seeded and transfected with lentiviruses containing GFP-LC3B (GenePharma) for 24 h, and the culture medium with lentiviruses was replaced. The MSCs were then treated with different drugs for 24 h. The punctuate pattern of LC3B in transfected cells was detected and analyzed immediately under a fluorescence microscope.

### Cell migration assay

Polycarbonate Membrane Transwell® Inserts (5.0 μm; Corning) were used to detect CD4^+^ T cell migration. A total of 2 × 10^4^ MSCs were seeded in the lower chamber of the Transwell and treated with 3-MA or rapamycin for 24 h. Then, the cells were washed thoroughly with PBS and added with 600 μL of new RPMI-1640 medium. After culturing for another 24 h, 2 × 10^5^ purified CD4^+^ T cells were added to the upper chamber with 100 μL of RPMI-1640 medium and placed into the Transwell. Moreover, only MSC culture supernatants were placed in the lower chambers in some experiments. In addition, 600 μL RPMI-1640 medium placed in the lower chamber without MSCs served as the blank control group. After co-culturing at 37 °C in 5% CO_2_ for 4 h, the CD4^+^ T cells that had migrated to the lower chamber were collected and counted by flow cytometry based on forward scatter (FSC) and side scatter (SSC) parameters.

### Quantitative real-time PCR

Quantitative real-time PCR (qRT-PCR) analysis was performed as described previously [9]. Briefly, RNA was isolated from cells using TRIzol (Invitrogen) and immediately converted to cDNA using a PrimeScriptTM RT Reagent Kit (TaKaRa). qRT-PCR was performed on a LightCycler 480 Real-Time PCR system (Roche) using SYBR Premix Ex Taq (TaKaRa). The expression level of the GAPDH gene was used to normalize mRNA expression for real-time PCR, and the relative expression levels of other relative genes were analyzed using the 2^−△△^ Ct method. The sequences for the primers used in this study are summarized in Additional file 6: Table S1.

### Western blot analysis

MSCs and CD4^+^ T cells from different groups were lysed, and protein was quantified as described previously. Equal amounts of protein were separated by 10% and 15% sodium dodecyl sulfate-polyacrylamide gel electrophoresis and transferred to polyvinylidene fluoride (PVDF) membranes (Millipore). The membranes were blocked with 5% non-fat milk in Tris-buffered saline containing Tween 20 for 1 h at room temperature and incubated overnight at 4 °C with the following primary antibodies: anti-LC3 (L7543; Sigma-Aldrich), anti-p62 (#8025; Cell Signaling Technology), anti-TGF-β1 (ab27969; Abcam), anti-CXCL8 (208-IL-010; R&D Systems), or anti-GAPDH. After washing three times, the PVDF membranes were incubated with the corresponding secondary antibodies (1:3000; Santa Cruz Biotechnology) for 1 h at room temperature. Specific antibody-antigen complexes were detected using Immobilon Western Chemiluminescent Horseradish Peroxidase Substrate (Millipore). The intensity of each band was determined using ImageJ software (version 1.49e).

### Enzyme-linked immunosorbent assay

The concentrations of CXCL8, prostaglandin E_2_ (PEG-2), interleukin 10 (IL-10), indoleamine 2, 3 (IDO), interleukin 6 (IL-6), and TGF-β1 in the cell culture supernatants were measured using Quantikine enzyme-linked immunosorbent assay kits for CXCL8, PEG-2, IL-10, IDO, and IL-6 (R&D Systems) and TGF-β1 (Sigma-Aldrich), respectively, according to the manufacturer’s instructions. It should be noted that the samples must be activated before using the Human TGF-β1 enzyme-linked immunosorbent assay (ELISA) Kit. All results were normalized to the total protein content.

### Cytometric bead array CBA

A CBA kit (BD Biosciences) was used to measure the cytokine levels in cell culture supernatants according to the manufacturer’s instructions. The concentration of each cytokine was determined according to the intensity of PE fluorescence relative to a standard curve. In addition, all results were analyzed with FCAP Array software.

### Lentivirus construction and infection

TGF-β1 cDNA (NM_000660.6) cDNA cloned into the lentivector-transferred plasmid pLVX-IRES-ZsGreen (Clontech Laboratories, Inc.) was obtained from GenePharma Co. and named Lenti-TGF-β1. Lentiviruses were generated by co-transfecting the packaging vectors psPAX2 and pMD2.G into 293T cells. The supernatants were collected, filtered, and concentrated after culturing for 72 h.

Lentiviruses encoding short hairpin (sh) RNA of TGF-β1 with a target sequence of 5′-GCAGAGTACACACAGCATATA-3′ (shTGF-β1) were generated by GenePharma Co. The sequence 5′-TTCTCCGAACGTGCACGTTTC-3′ was used as a negative control (shNC). pGLVH1/GFP/Puro (Gene Pharma) and packing plasmids (pGag/Pol, pRev, and pVSV-G) were transfected into 293T cells to produce lentiviruses. The culture supernatants containing lentiviruses were filtered and concentrated at 3 days after transfection.

Transfection of MSCs was performed by incubating them in medium containing the lentivirus and Polybrene (5 μg/ml) for 24 h at a multiplicity of infection (MOI) of 50. Related experiments were performed later.

### Statistical analysis

All data are expressed as the mean ± standard deviations (SDs). Statistical analysis was performed using one-way analysis of variance (ANOVA) followed by the Bonferroni test with SPSS (version 13.0; SPSS, Inc.). *P* < 0.05 was considered statistically significant.

## Results

### Autophagy regulates MSC-mediated CD4^+^ T cell recruitment

All MSCs used in our studies expressed classic MSC phenotypes (Additional file 1: Figure S1A) and showed tri-lineage differentiation potential (Additional file 1: Figure S1B). As previously reported, we used 3-MA (an autophagy inhibitor) and rapamycin (an autophagy inducer) to regulate autophagy in MSCs [9]. In addition, we reconfirmed that 3-MA inhibited autophagy but rapamycin activated autophagy, as detected by western blot (Additional file 2: Figure S2A) and GFP-LC3B (Additional file 2: Figure S2B). Furthermore, CCK-8 revealed that the dose of 3-MA and rapamycin used in this study did not affect cell proliferation (Additional file 2: Figure S2C). Meanwhile, there was no difference in CD4^+^ T cell autophagy and apoptosis among groups (Additional file 3: Figure S3A, B).

**Fig. 1:**
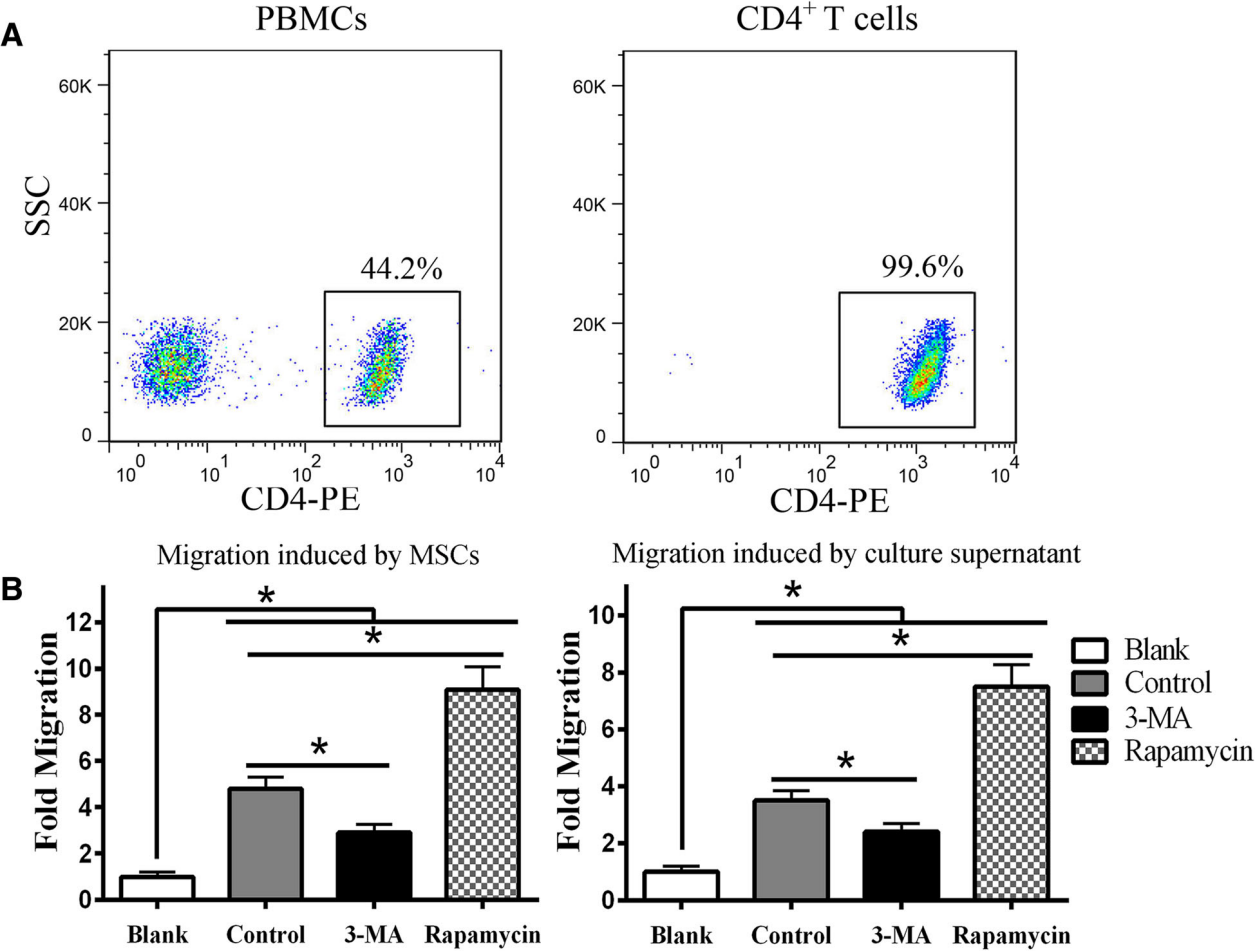
Autophagy regulates MSC-mediated CD4^+^ T cell recruitment. **a** CD4^+^ T cells were purified from PBMCs and the purity was assessed by flow cytometry. **b** After co-culturing with MSCs for 4 h, the number of migrated CD4^+^ T cells was analyzed by flow cytometry. 3-MA weakened MSC-mediated migration, while rapamycin enhanced migration compared with the control group without treatment. The MSC culture supernatants showed similar results. Statistical significance among groups was determined by using SPSS. Values are presented as the means ± SD of 18 samples per group. * indicates *P* < 0.05. (Only growth medium without MSCs was used in the blank group)

To harvest the CD4^+^ T cells, PBMCs were separated using Ficoll-Hypaque gradient centrifugation, and CD4^+^ T cells were isolated using magnetic beads. The CD4^+^ T cell purity was assessed by flow cytometry using an anti-CD4^+^ T cell antibody. The positive rate of CD4^+^ T cells after purification was 99.6%, whereas that of PBMCs was just 44.2% (Fig. 1a).

Then, an in vitro Transwell co-culture system was used to test whether MSC autophagy affected CD4^+^ T cell migration to MSCs. After co-culture for 4 h, the number of CD4^+^ T cells that had migrated to the lower chamber was counted by flow cytometry based on FSC and SSC parameters. Interestingly, the results showed that 3-MA weakened MSC-mediated CD4^+^ T cell migration, while rapamycin remarkably enhanced the migration compared to the untreated control group. In addition, the MSC culture supernatants collected from different groups showed the similar results except that less migration was observed than in the MSC groups (Fig. 1b). Furthermore, the MSC and supernatant groups recruited many more CD4^+^ T cells than the blank group, which suggested that the CD4^+^ T cell migration observed in our study was mainly directed but random. In summary, the MSC recruitment of CD4^+^ T cells might depend on soluble chemokines, suggesting that CD4^+^ T cell migration can be regulated by controlling MSC autophagy.

### Autophagy enhances MSC-mediated CD4^+^ T cell recruitment through CXCL8

**Fig. 2:**
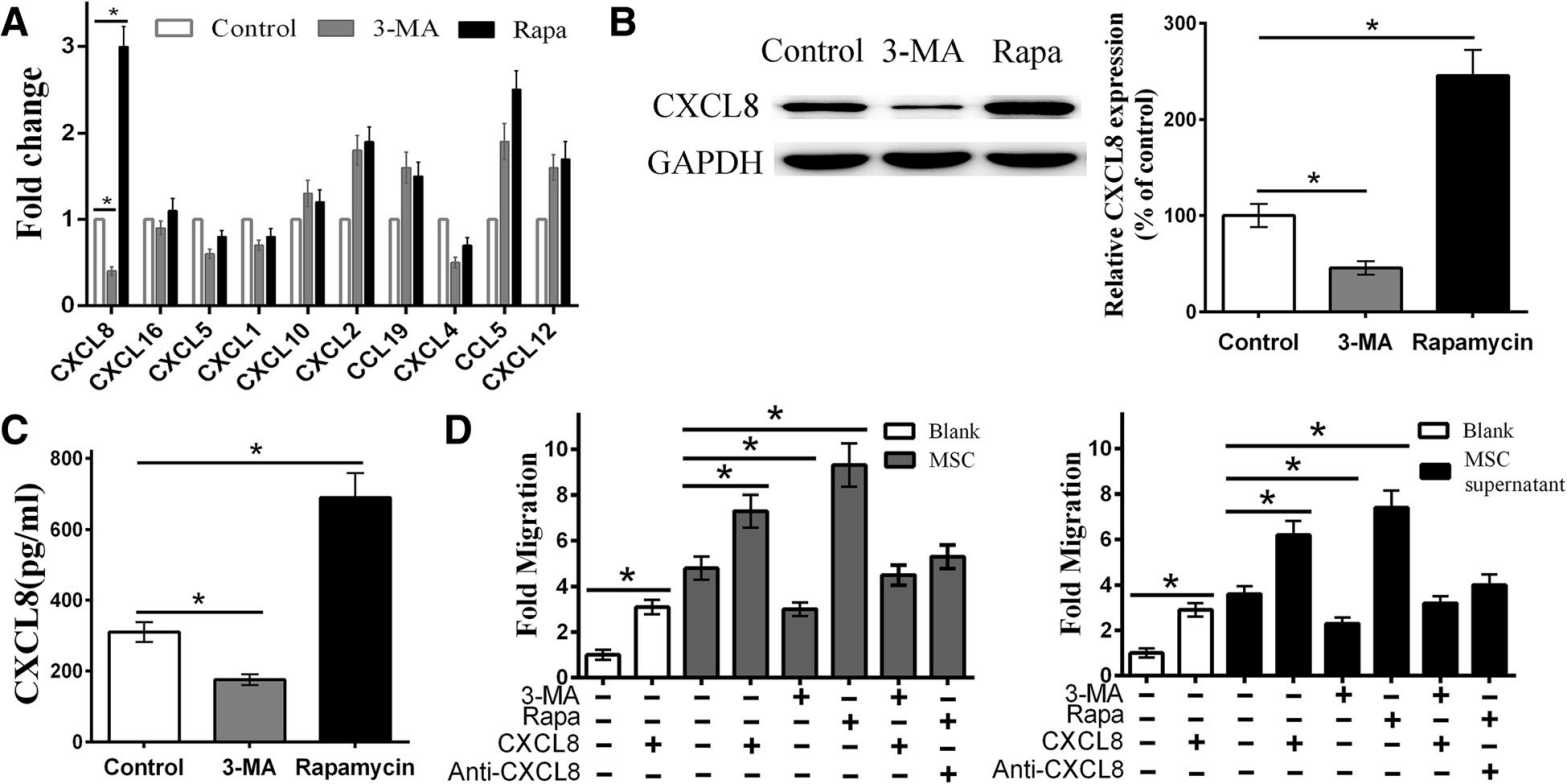
Autophagy increases MSC-mediated CD4^+^ T cell migration through CXCL8. **a** After treatment with different drugs for 24 h, qRT-PCR showed that 3-MA could downregulate while rapamycin could upregulate the CXCL8 expression, indicated that autophagy could regulate the expression of CXCL8 but not other chemokines. **b**, **c** Western blot and ELISA were used to confirm the protein expression/secretion of CXCL8 among different groups, which was decreased in the 3-MA group but increased in the rapamycin group. **d** Exogenous CXCL8 increased CD4^+^ T cell recruitment in the blank, control, and 3-MA groups, while exogenous anti-CXCL8 antibody decreased recruitment in the rapamycin group. The MSCs and MSC culture supernatant showed similar results. All the CD4^+^ T cell migration was detected by flow cytometry. Values are presented as the means ± SD of 18 samples per group. * indicates *P* < 0.05

As we speculated, chemokines may play an important role in MSC-mediated CD4^+^ T cell migration. Therefore, we analyzed the recognized, important, and highly expressed chemokines in MSCs, such as CXCL8, CXCL1, CXCL10, and CXCL12. qRT-PCR analysis showed a positive correlation between MSC autophagy and CXCL8 mRNA expression (Fig. 2a). We then confirmed the protein levels of CXCL8 among different groups using western blotting and ELISA. Similar to the qRT-PCR results, the 3-MA-pretreated group expressed and secreted less CXCL8, while the rapamycin-pretreated group showed the opposite result for the MSC and the cell culture supernatant groups (Fig. 2b, c).

To confirm our hypothesis further, exogenous CXCL8 was added to the lower chamber of the blank, control, and 3-MA groups, and exogenous anti-CXCL8 antibody was added to the lower chamber of the rapamycin group. The results demonstrated that exogenous CXCL8 increased CD4^+^ T cell recruitment in the CXCL8 alone, MSCs+CXCL8, and 3-MA+CXCL8 groups in a dose-dependent manner. The results revealed that the CXCL8 alone and MSCs+CXCL8 groups showed a higher CD4^+^ T cell recruitment than the blank and control groups respectively, when the exogenous CXCL8 concentration reached 150 ng/mL. CD4^+^ T cell migration in 3-MA-pretreated MSCs was similar to that in control MSCs. In addition, increasing the concentration of the anti-CXCL8 antibody gradually reversed the strong CD4^+^ T cell recruitment in the rapamycin group. When a concentration of 350 ng/mL antibody was added, the number of CD4^+^ T cells migrating towards rapamycin-pretreated MSCs was significantly reduced, but remained slightly higher than that observed in the control group. Besides, the CXCL8 alone group showed a higher CD4^+^ T cell recruitment than the blank group. In addition, we obtained a similar result in the MSC culture supernatant group (Fig. 2d). These results suggest that CXCL8 plays a key role in MSC-mediated CD4^+^ T cell recruitment in our study; however, other chemokines may also be involved. In summary, we suggest that autophagy can regulate MSC-mediated CD4^+^ T cell recruitment through CXCL8.

### MSC autophagy increases the ratio of Treg cells

**Fig. 3:**
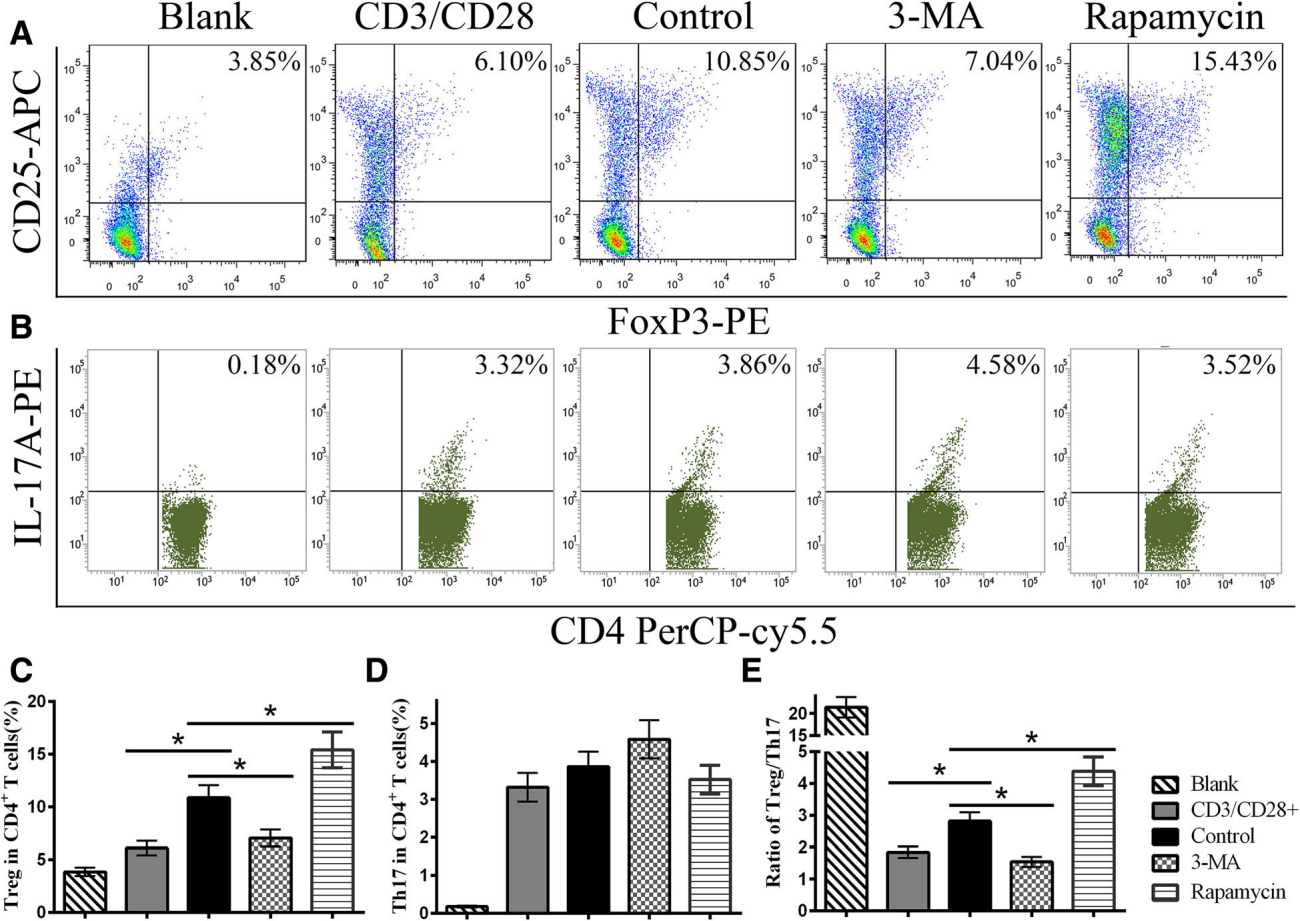
MSC autophagy increases the ratio of Treg cells. Nonactivated CD4^+^ T cells were used as the blank group. **a**, **c** The flow cytometry results revealed the ratio of Treg cells decreased in the 3-MA group but increased in the rapamycin group compared with the control group without treatment. **b**, **d** There were no differences in the Th17 ratio among groups. **e** The change in the Treg/Th17 ratio was similar to the change in Treg ratio among the control and MSC groups. Values are presented as the means ± SD of 18 samples per group. * indicates *P* < 0.05

MSCs exert certain functions in the polarization of CD4^+^ T cells. To detect whether autophagy affected MSC-induced CD4^+^ T cell differentiation, flow cytometry was used to analyze CD4^+^ T cell subsets. When CD3/CD28-treated CD4^+^ T cells were co-cultured with MSCs, the ratio of Treg cells was increased, but the ratio of Th17 cells was slightly decreased. Moreover, 3-MA-pretreated MSCs decreased the ratio of Treg cells, while rapamycin-pretreated MSCs increased the ratio of Treg cells compared with the control group (Fig. 3a, c). However, few significant differences in the Th17 ratio were seen among the MSC groups (Fig. 3b, d). In addition, the Treg/Th17 ratio of the 3-MA group was lower than that of the control group but a significantly higher ratio was observed in the rapamycin group (Fig. 3e).

### MSC autophagy decreases the ratio of Th1 cells

**Fig. 4:**
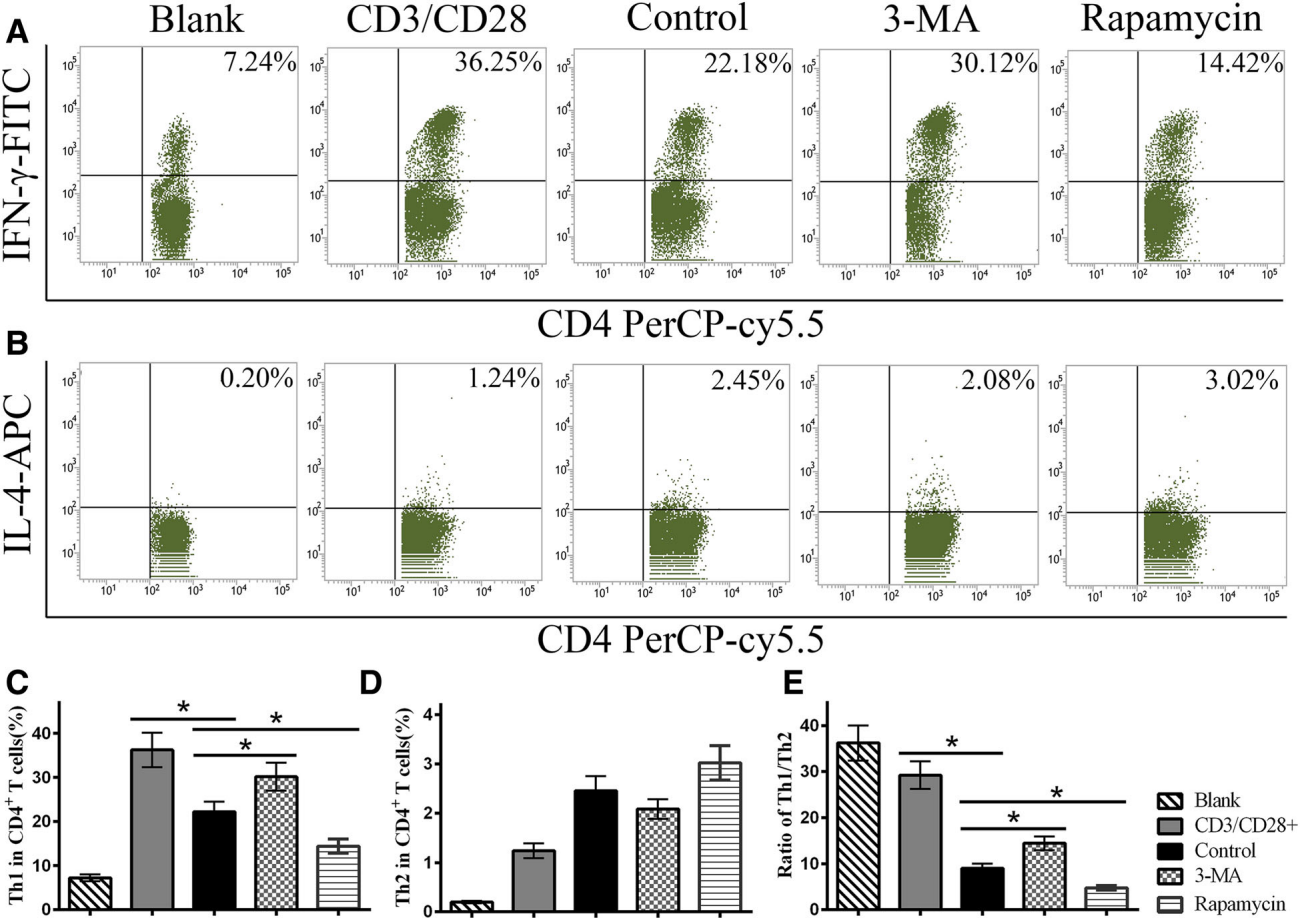
MSC autophagy decreases the ratio Th1 cells. Flow cytometry was used to detect Th1 and Th2 cells in the co-culture systems. **a**, **c** MSCs remarkably inhibited Th1 cell differentiation. The 3-MA group showed a higher ratio of Th1 cells while the rapamycin group showed a lower ratio of Th1 cells than the control group. **b**, **d** The differences in Th2 cells among MSC groups were not statistically significant. **e** The change in the Th1/Th2 ratio was similar to the change in the Th1 ratio among the control and MSC groups. Values are presented as the means ± SD of 18 samples per group. * indicates *P* < 0.05

At the end of the co-culture, the ratio of Th1 cells in the CD3/CD28^+^ group was remarkably elevated, and MSCs effectively inhibited Th1 cell differentiation. Further analysis showed that the 3-MA group had a higher ratio of Th1 cells but the rapamycin group had a lower ratio of Th1 cells compared with the control MSC group (Fig. 4a, c). This finding suggests that autophagy might strengthen the ability of MSCs to suppress Th1 cell polarization. Meanwhile, the ratio of Th2 cells was slightly increased in the co-culture system, but the differences among the MSC groups were not statistically significant (Fig. 4b, d). Thus, the ratio of Th1/Th2 cells was increased in the 3-MA group but decreased in the rapamycin group compared with the control group (Fig. 4e).

### Changes in the levels of IL-17A, interferon-γ, and IL-2 cytokines among groups

**Fig. 5:**
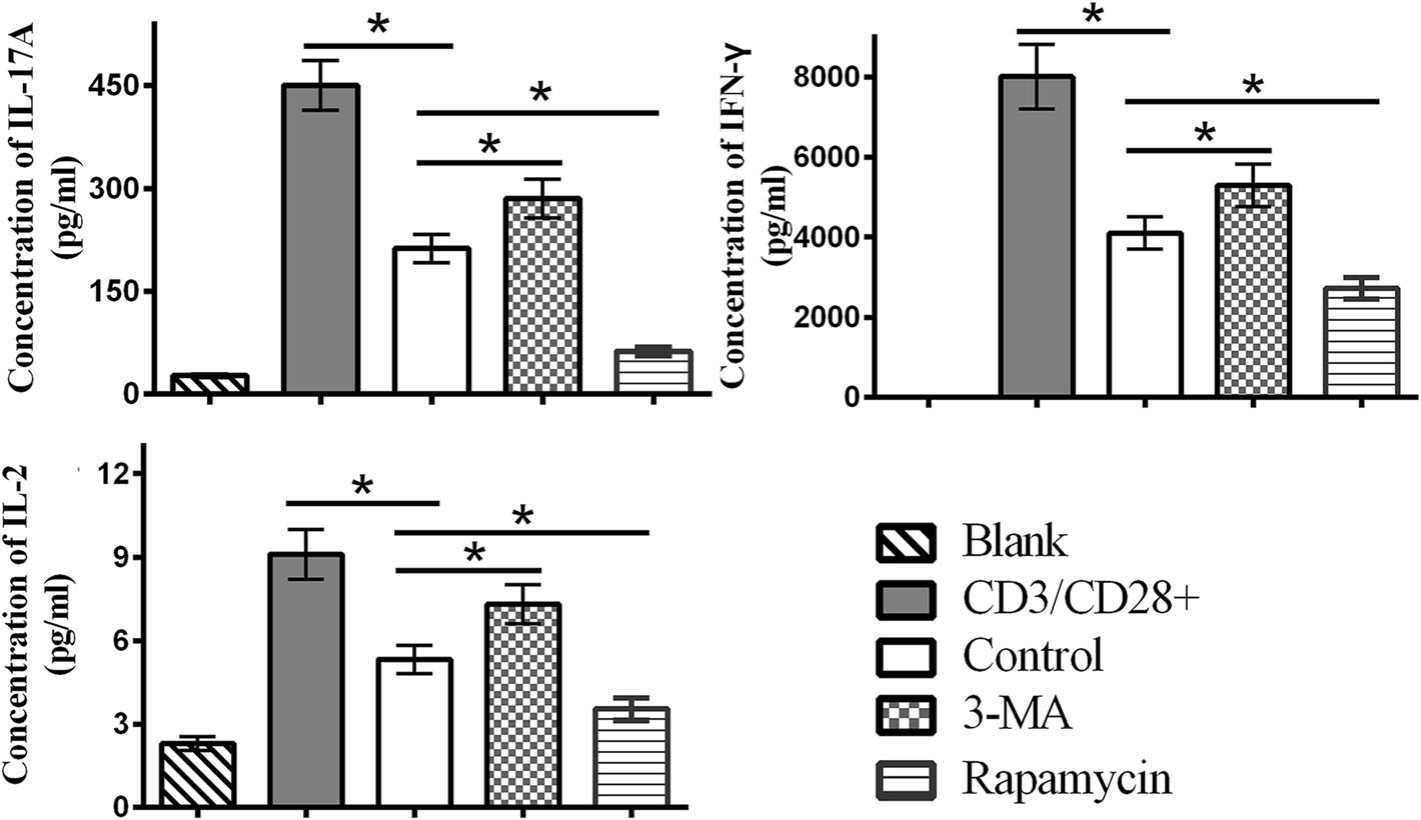
Autophagy decreases the inflammatory cytokines in co-culture. On the 5th day of co-culture, a CBA kit was used to measure the levels of cytokines in the co-culture system. IL-17A, IFN-γ, and IL-2 increased in the 3-MA-pretreated group but decreased in the rapamycin-pretreated group compared with the control group. The results suggested that autophagy of MSCs could regulate the secretion of the inflammatory cytokines in co-culture. Values are presented as the means ± SD of 18 samples per group. * indicates *P* < 0.05

A CBA kit was used in our study to measure the levels of cytokines in the co-culture system. Our results demonstrated that IL-17A, interferon (IFN)-γ, and IL-2 were increased in the 3-MA-pretreated group, but were decreased in the rapamycin-pretreated group compared with the control group (Fig. 5). There were no obvious differences in tumor necrosis factor-α, IL-10, IL-6, and IL-4 levels among the MSC groups (data not shown). These results suggest that autophagy regulated various cytokines, including IL-17A, IFN-γ, and IL-2, in the co-culture system, which indicated that MSC autophagy could alleviate the inflammation.

### TGF-β1 reverses the effect of autophagy on MSC-mediated CD4^+^ T cell differentiation

**Fig. 6:**
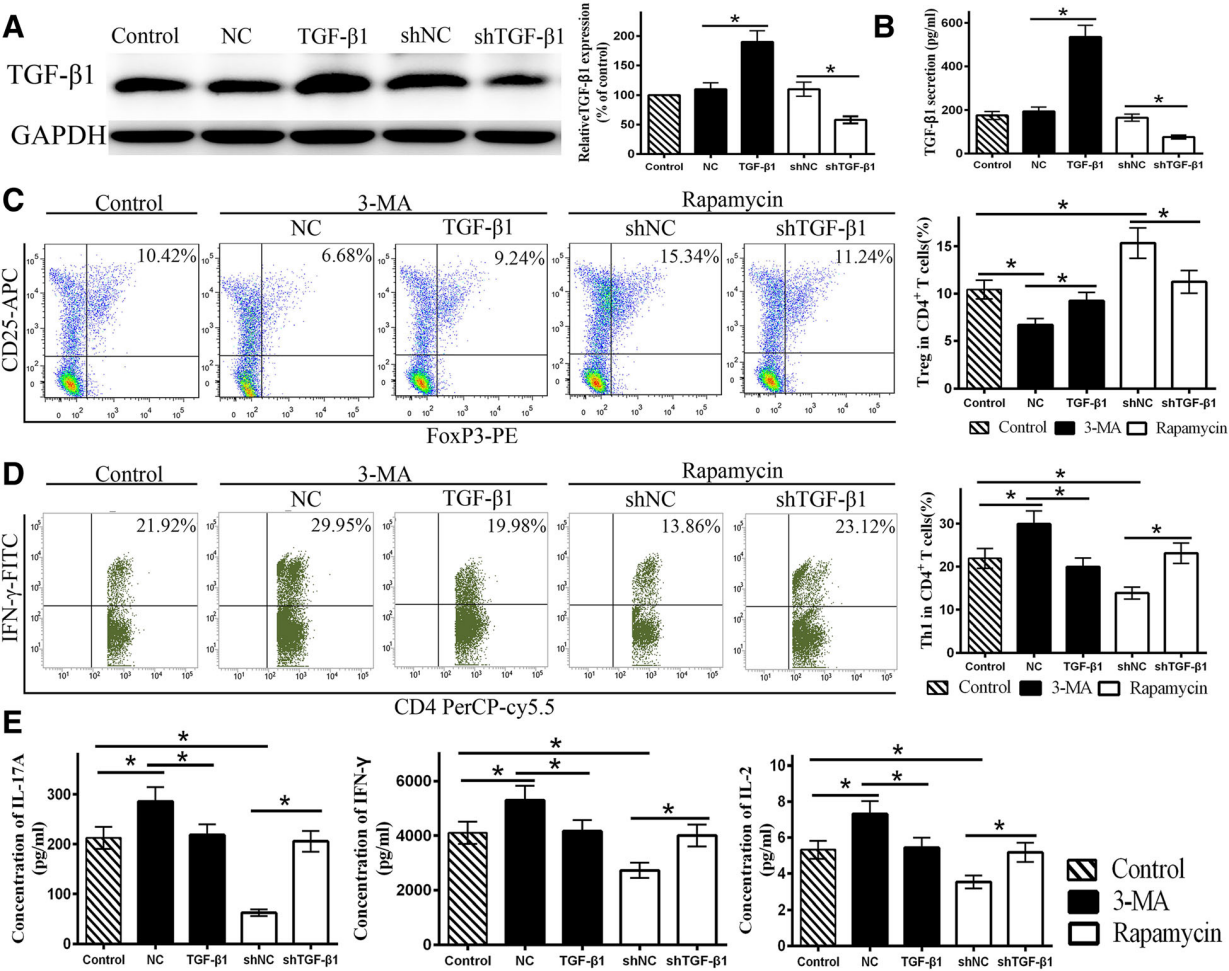
TGF-β1 reverses the effect of autophagy on MSC-mediated CD4^+^ T cell differentiation. **a**, **b** Lentiviruses were used to diminish and overexpress TGF-β1 in MSCs. TGF-β1 overexpression was defined as TGF-β1 (NC, negative control), and TGF-β1 knockdown was defined as shTGF-β1 (shNC, negative control). The western blotting and ELISA results showed that TGF-β1 overexpression increased MSC TGF-β1 protein level and secretion, while shTGF-β1 exerted the opposite result. **c** The flow cytometry results showed that TGF-β1 overexpression increased the Treg ratio in the 3-MA group, and shTGF-β1 decreased the Treg ratio in the rapamycin group. **d** TGF-β1 overexpression decreased the ratio of Th1 cells in the 3-MA group, and shTGF-β1 increased the ratio of Th1 cells in the rapamycin group. **e** The CBA results indicated TGF-β1 overexpression reduced the inflammatory cytokines (IL-17A, IFN-γ, and IL-2) in the 3-MA group, while shTGF-β1 increased them in the rapamycin group. Values are presented as the means ± SD of 18 samples per group. * indicates *P* < 0.05

We previously reported that autophagy regulated the expression and secretion of TGF-β1 by MSCs [9], which is as an immunoregulatory molecule. We confirmed this result in this study, but the other important cytokines concluding PGE-2, IL-10, IDO, and IL-6 show no correlation with the MSC autophagy (Additional file 4: Figure S4A), Thus, we speculated that TGF-β1 might play a key role in regulating CD4^+^ T cell differentiation. We found that TGF-β1 overexpression or shTGF-β1 effectively altered TGF-β1 expression at both the gene and protein levels (Fig. 6a). In addition, similar results were observed for the MSC culture supernatant (Fig. 6b). Flow cytometry demonstrated that TGF-β1 overexpression increased the ratio of Treg cells in both the control and 3-MA groups such that no obvious differences were observed between them. Furthermore, shTGF-β1 eliminated the differences between the rapamycin and control groups (Fig. 6c). Similarly, TGF-β1 overexpression enhanced the inhibition of Th1 cells in the 3-MA group, and shTGF-β1 reduced the Th1 cell ratio in the rapamycin group (Fig. 6d). Overexpression of TGF-β1 and transfection with shTGF-β1 did not affect the number of Th17 and Th2 cells among the groups. Thus, TGF-β1 overexpression increased the ratio of Treg/Th17 cells and decreased the ratio of Th1/Th2 cells in the 3-MA group, while shTGF-β1 induced the opposite effect in the rapamycin group. Furthermore, we observed extremely high secretion of IL-6, which show the same trend as MSC autophagy, even though there was no statistically significant difference among the groups. Thus, exogenous IL-6 protein and neutralizing antibody were added into the 3-MA and rapamycin groups respectively, to assess the role of IL-6 on the polarization of CD4^+^ T cells. The results revealed that IL-6 and neutralizing antibody had a slight effect on the polarization of Treg and Th1 cells (Additional file 4: Figure S4B). In summary, these results suggest that MSC autophagy can regulate CD4^+^ T cell differentiation by the secretion of TGF-β1. Furthermore, Lv-TGF-β1 increased IL-17A, IFN-γ, and IL-2 levels in the rapamycin group, while TGF-β1 overexpression reduced the levels of these cytokines in the 3-MA group (Fig. 6e). Taken together, these data suggest that autophagy in MSC could play a protective role in immune reactions.

## Discussion

In this study, we first demonstrated that autophagy in MSC increased CD4^+^ T cell migration mediated by CXCL8. We also found that MSC autophagy increased the ratio of Treg cells, but decreased the ratio of Th1 cells and reduced proinflammatory cytokines in the co-culture system. Overexpressing or inhibiting TGF-β1 rectified the differences in CD4^+^ T cell polarization and proinflammatory cytokines among groups. Altogether, our results indicate that autophagy regulated MSC-mediated immunoregulatory functions, including CD4^+^ T cell migration and polarization.

MSCs are multipotent progenitors that can be isolated from the bone marrow, adipose tissue, and many other tissues [17, 18]. MSCs exhibit immunomodulatory properties and low immunogenicity and have been applied successfully for the treatment of many diseases such as systemic lupus erythematosus (SLE) and graft-versus-host disease [19–21]. However, the underlying mechanism remains elusive. Some researchers demonstrated that proinflammatory cytokines might improve the MSC-mediated therapy because it may affect the immunomodulatory of MSCs [22]. Interestingly, some studies indicated that the inflammatory factors also induce MSC autophagy. Autophagy is a basic cellular homeostatic process involved in various functions such as apoptosis [23] and differentiation of MSCs [24]. In addition, we previously reported that MSC autophagy could improve the immunosuppression of CD4^+^ T cells. However, whether autophagy plays an important role in other aspects of MSC-mediated immunomodulation remains unknown and requires further study.

To explore this question, we sought to detect the effects of MSC autophagy on CD4^+^ T cell migration and polarization. CD4^+^ T cells are crucial for directing appropriate immune responses and involved in disease progression in autoimmune diseases and other diseases [25, 26]. Several studies have demonstrated that MSCs can recruit and modulate the functions of CD4^+^ T cells [27–30]. Thus, detecting CD4^+^ T cell recruitment and differentiation can be used to assess the efficiency of MSC-mediated therapy. Some studies hold that excessive lymphocyte migration can aggravate local inflammation and the progression of some diseases [31, 32]. However, other studies have suggested that directed immunocyte migration plays a protective role in physiological and pathophysiological conditions. Immunocytes can migrate to the target, playing an important role in the immune response, but their migration is random without the guidance of chemokines. Some studies defined CXCL8 as a “signpost” for various immunocytes [33]. Interestingly, our results showed that MSC autophagy strengthened CD4^+^ T cell migration through CXCL8. Therefore, we speculated that autophagy enhanced the signpost effect of MSCs mediated by CXCL8, which might promote immune responses, intracellular pathogen clearance, etc. In addition, we also confirmed that autophagy could reduce inflammation and promote anti-inflammation. In summary, we conclude that MSC-mediated CD4^+^ T cell recruitment enhanced by autophagy plays a protective role.

CD4^+^ T lymphocytes can differentiate into distinct effector Th subsets (Th1, Th2, and Th17 cells) and Treg cells, which produce different cytokines with immune regulatory functions [34–36]. Th1 and Th17 cells are recognized as proinflammatory, while Th2 and Treg cells are regarded as anti-inflammatory. Our experiments showed that MSC autophagy upregulated the ratio of Treg cells and downregulated the ratio of Th1 cells. Moreover, it is worth mentioning that the rapamycin group exhibited a more remarkable change, which was consistent with the change in the level of autophagy. Similarly, some in vivo experiments also suggested that rapamycin augments the immunomodulatory of MSCs [37, 38]. These results suggest that autophagy can enhance MSC-mediated suppression on proinflammatory responses by reducing inflammation and promoting anti-inflammatory activity.

Upon further analysis, we found that the ratio of Th1 cells among CD4^+^ T cells was highest, and the change in Th1 cells was most obvious when co-cultured with MSCs. Th1 cells have been recognized as an important part of the immune system because they are responsible for eliminating invading pathogens [39, 40]. However, if the Th1 cell response is not effectively regulated, the progression of various diseases may become aggravated [41, 42]. Consistent with our results, numerous studies have demonstrated that MSCs can suppress the Th1 subset [43], which might help to explain why MSC-mediated therapy is effective.

Some questions remain to be answered, such as the mechanism of how autophagy affects MSC-mediated immunomodulation. Previous studies indicated that paracrine factors such as IDO, prostaglandin E_2_, and COX_2_ and cell-cell contacts play important roles in MSC-mediated immunomodulation [44]. In addition, we previously found that autophagy regulates TGF-β1 secretion by MSCs [9], but other cytokines had no relevance with MSC autophagy. The present study showed that overexpression or inhibition of TGF-β1 eliminated the differences in CD4^+^ T cell polarization among groups. However, modulating TGF-β1 could not completely reverse the discrepancies in the 3-MA and rapamycin groups compared with the control group, which suggests that other factors also participated in this process. Thus, we conclude that TGF-β1 plays a key role in the autophagy-regulated MSC immunomodulation, and therefore, TGF-β1 may be a novel target for improving MSC-mediated therapy.

In this study, we demonstrated MSC autophagy increased CD4^+^ T cell migration through CXCL8 and promoted Treg cell differentiation but inhibited Th1 cell differentiation by secreting TGF-β1. These results contribute to a better understanding of the underlying mechanism of MSC-mediated therapy and suggest a potential new strategy for improving this treatment. However, there are some limitations of this study that should be considered. It is unknown whether these results will be corroborated in vivo. Moreover, it is unclear whether MSC autophagy has analogous effects on other immunocytes such as B cells, natural killer cells, and neutrophils. Further studies should be devoted to exploring these questions and developing an effective and safe treatment.

## Conclusion

Our study demonstrates that autophagy could regulate the migration and polarization of CD4^+^ T cells by MSCs through affecting CXCL8 and TGF-β1, respectively. These results provide a potential new strategy for improving MSC-mediated therapy.

## Additional files

Additional file 1: **Figure S1.** Identification of MSCs. (A) The flow cytometric results: MSCs were positive for CD29, CD44, and CD105 but negative for HLA-DR, CD34 and CD45. (B) MSCs were plastic-adherent and spindle-shaped. The multipotent differentiation potential of MSCs was verified by inducing differentiation and staining. After induction for indicated days, osteogenesis, adipogenesis and chondrogenesis were stained by Alizarin Red S, Oil Red O and Toluidine blue staining respectively. MSCs and Osteogenesis (× 40, scale bar = 500 μm), adipogenesis (× 100, scale bar = 200 μm) and chondrogenesis (× 100, scale bar = 200 μm) were observed by microscopy. (TIF 9928 kb)

Additional file 2: **Figure S2.** Regulation of the autophagy of MSCs. (A) MSCs were pretreated with 3-methyladenine (3-MA) and rapamycin for 24 h, and autophagy was then assessed by western blot analysis for LC3-II and P62. The results showed that 3-MA decreased while rapamycin increased the autophagy of MSCs. Values are presented as the means± SD of 18 samples per group. * indicates *P* < 0.05. (B) MSCs were transfected with GFP-LC3B lentiviruses for 24 h and treated with 3-MA (10 mM) and rapamycin (3 μM). The puncta staining was then observed by fluorescence microscope. The green puncta were obviously reduced in cells treated with 3-MA but increased in cells treated with rapamycin (× 200, scale bar = 50 μm). (C) The CCK-8 result showed there was no difference in the proliferation of MSCs treated with different drugs. (TIF 8117 kb)

Additional file 3: **Figure S3.** No difference in apoptosis and autophagy of CD4^+^ T cells in co-culture system among groups can be observed. (A) The autophagy of CD4^+^ T cells from the co-culture system of three groups was assessed by western blot analysis for LC3-II and P62. The results revealed no difference of the CD4^+^ T cell autophagy among groups. (B) Flow cytometry was used to detect the apoptosis of CD4^+^ T cells, no discrepancies were observed in the three groups. Values are presented as the means ± SD of 18 samples per group. * indicates *P* < 0.05. (TIF 6728 kb)

Additional file 4: **Figure S4.** The secretion patterns of the immunoregulatory factors in MSCs are affected by autophagy. (A) The secretion levels of TGF-β1, PEG-2, IL-10, IDO and IL-6 in MSCs were measured by using Elisa. Only TGF-β1 secretion showed a positive correlation with MSC autophagy. (B) Exogenous IL-6 protein and antibody were added to the 3-MA and rapamycin group respectively to assessed its role in the MSC-mediated CD4^+^ T cell polarization. The flow cytometry results revealed that exogenous IL-6 protein or anti-IL-6 antibody exerted little effect on the MSC-mediated Treg and Th1 cell polarization. Values are presented as the means ± SD of 18 samples per group. * indicates *P* < 0.05. (TIF 9965 kb)

Additional file 5: **Figure S5.** DMSO does not exert visible effects on MSCs compared with the control group. (A) The number of migrated CD4^+^ T cells was analyzed by flow cytometry. DMSO did not affect the MSC-mediated CD4^+^ T cell migration, and the DMSO-pretreated MSC culture supernatants showed similar CD4^+^ T cell migration compared with the control group. (B) The expression and secretion of CXCL8 and TGF-β1 were similar between the DMSO-pretreated and control group. There was no difference in autophagy between DMSO-pretreated and control group, detected by western blotting targeting LC3 and P62. (C) The flow cytometry showed that DMSO did not affect the MSC-mediated Treg and Th1 polarization. Values are presented as the means ± SD of 18 samples per group. * indicates *P* < 0.05. (TIF 8346 kb)

Additional file 6: **Table S1.** Primers used for qRT-PCR (DOCX 16 kb)

## Abbreviations

3-MA: 3-Methyladenine
ANOVA: One-way analysis of variance
CBA: Cytometric Bead Array
CCK-8: Cell Counting Kit-8
CXCL8: C-X-C motif chemokine ligand 8
ELISA: Enzyme-linked immunosorbent assay kit
FSC: Forward scatter
GVHD: Graft-versus-host disease
IDO: Indoleamine 2, 3
IFN-γ: Interferon-γ
IL-10: Interleukin 10
IL-6: Interleukin 6
IL-8: Interleukin 8
MSCs: Mesenchymal stem cells
PBMCs: Peripheral blood mononuclear cells
PBS: Phosphate-buffered saline
PEG-2: Prostaglandin E_2_
PVDF: Polyvinylidene fluoride
qRT-PCR: Real-time quantitative reverse transcription-polymerase chain reaction
SDs: Standard deviations
SLE: Systemic lupus erythematosus
SSC: Side scatter
TGF-β1: Transforming growth factor-β1
Th1 cells: T helper 1 cells
Treg cells: Regulatory T cells
