# Supplementary figures and images for: Autophagy enhances mesenchymal stem cell-mediated CD4+ T cell migration and differentiation through CXCL8 and TGF-β1
Source: Stem Cell Res Ther. 2019 Aug 23;10:265. doi: 10.1186/s13287-019-1380-0 (PMC6708254; doi:10.1186/s13287-019-1380-0)

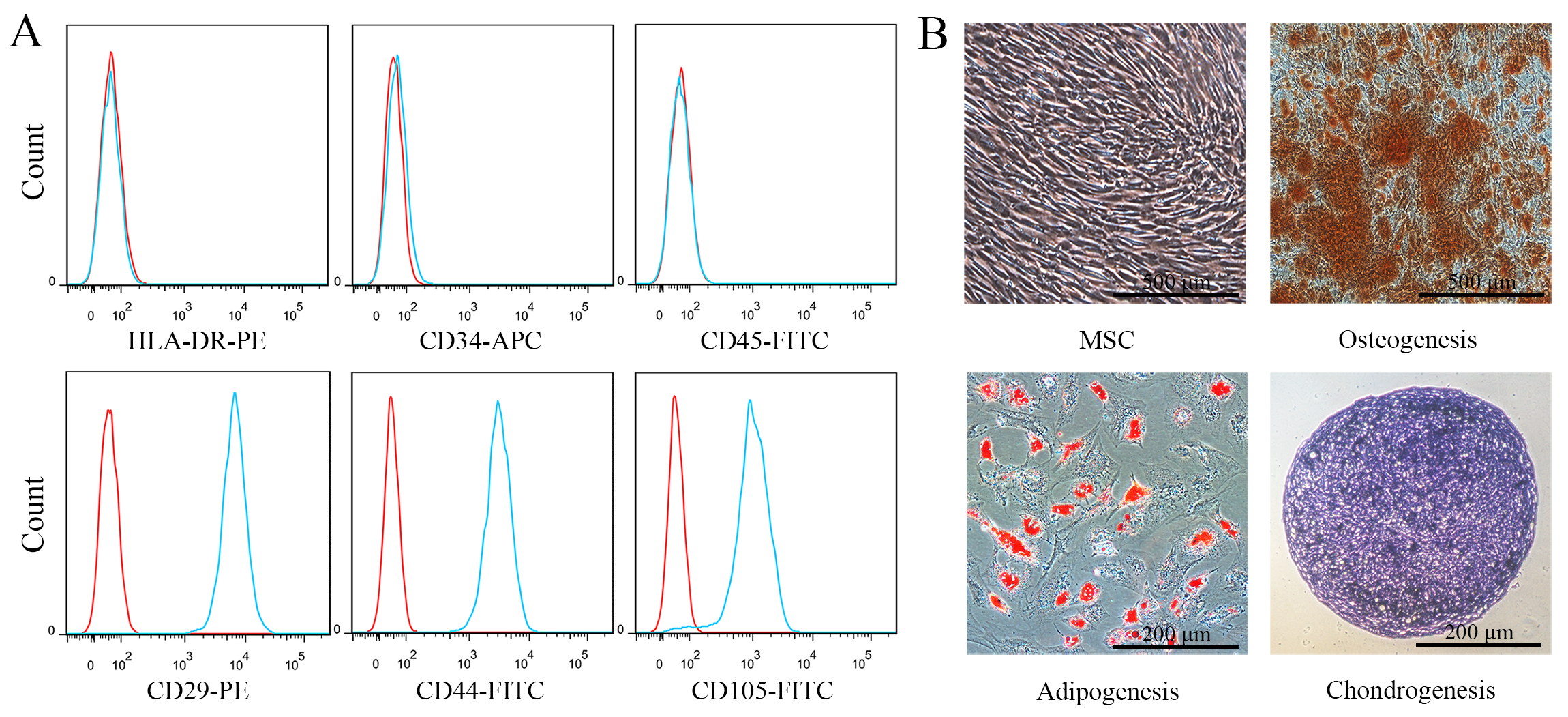

Supplement: Supplementary file 1 — Figure S1. Identification of MSCs. (A) The flow cytometric results: MSCs were positive for CD29, CD44, and CD105 but negative for HLA-DR, CD34 and CD45. (B) MSCs were plastic-adherent and spindle-shaped. The multipotent differentiation potential of MSCs was verified by inducing differentiation and staining. After induction for indicated days, osteogenesis, adipogenesis and chondrogenesis were stained by Alizarin Red S, Oil Red O and Toluidine blue staining respectively. MSCs and Osteogenesis (× 40, scale bar = 500 μm), adipogenesis (× 100, scale bar = 200 μm) and chondrogenesis (× 100, scale bar = 200 μm) were observed by microscopy. (TIF 9928 kb) [file 13287_2019_1380_MOESM1_ESM.tif]

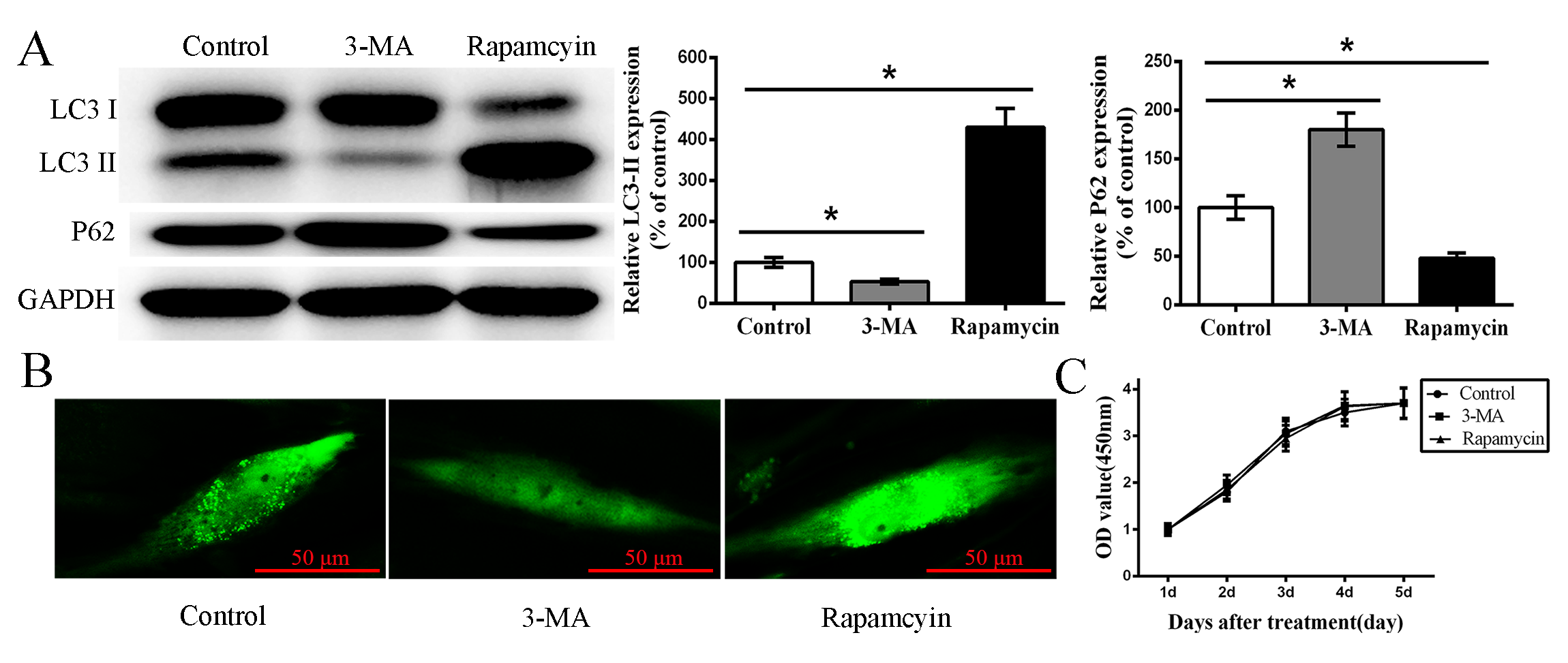

Supplement: Supplementary file 2 — Figure S2. Regulation of the autophagy of MSCs. (A) MSCs were pretreated with 3-methyladenine (3-MA) and rapamycin for 24 h, and autophagy was then assessed by western blot analysis for LC3-II and P62. The results showed that 3-MA decreased while rapamycin increased the autophagy of MSCs. Values are presented as the means± SD of 18 samples per group. * indicates P < 0.05. (B) MSCs were transfected with GFP-LC3B lentiviruses for 24 h and treated with 3-MA (10 mM) and rapamycin (3 μM). The puncta staining was then observed by fluorescence microscope. The green puncta were obviously reduced in cells treated with 3-MA but increased in cells treated with rapamycin (× 200, scale bar = 50 μm). (C) The CCK-8 result showed there was no difference in the proliferation of MSCs treated with different drugs. (TIF 8117 kb) [file 13287_2019_1380_MOESM2_ESM.tif]

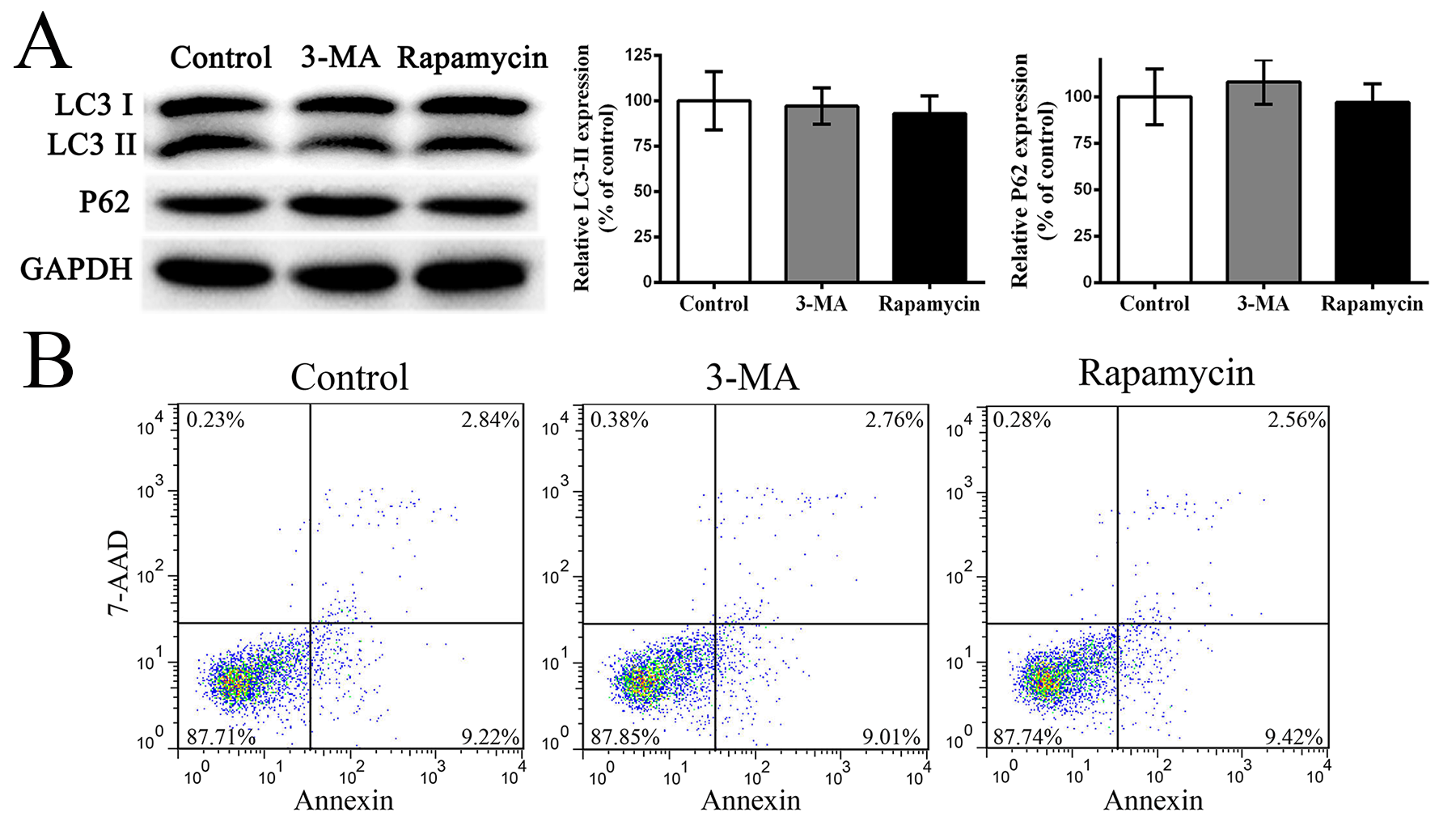

Supplement: Supplementary file 3 — Figure S3. No difference in apoptosis and autophagy of CD4+ T cells in co-culture system among groups can be observed. (A) The autophagy of CD4+ T cells from the co-culture system of three groups was assessed by western blot analysis for LC3-II and P62. The results revealed no difference of the CD4+ T cell autophagy among groups. (B) Flow cytometry was used to detect the apoptosis of CD4+ T cells, no discrepancies were observed in the three groups. Values are presented as the means ± SD of 18 samples per group. * indicates P < 0.05. (TIF 6728 kb) [file 13287_2019_1380_MOESM3_ESM.tif]

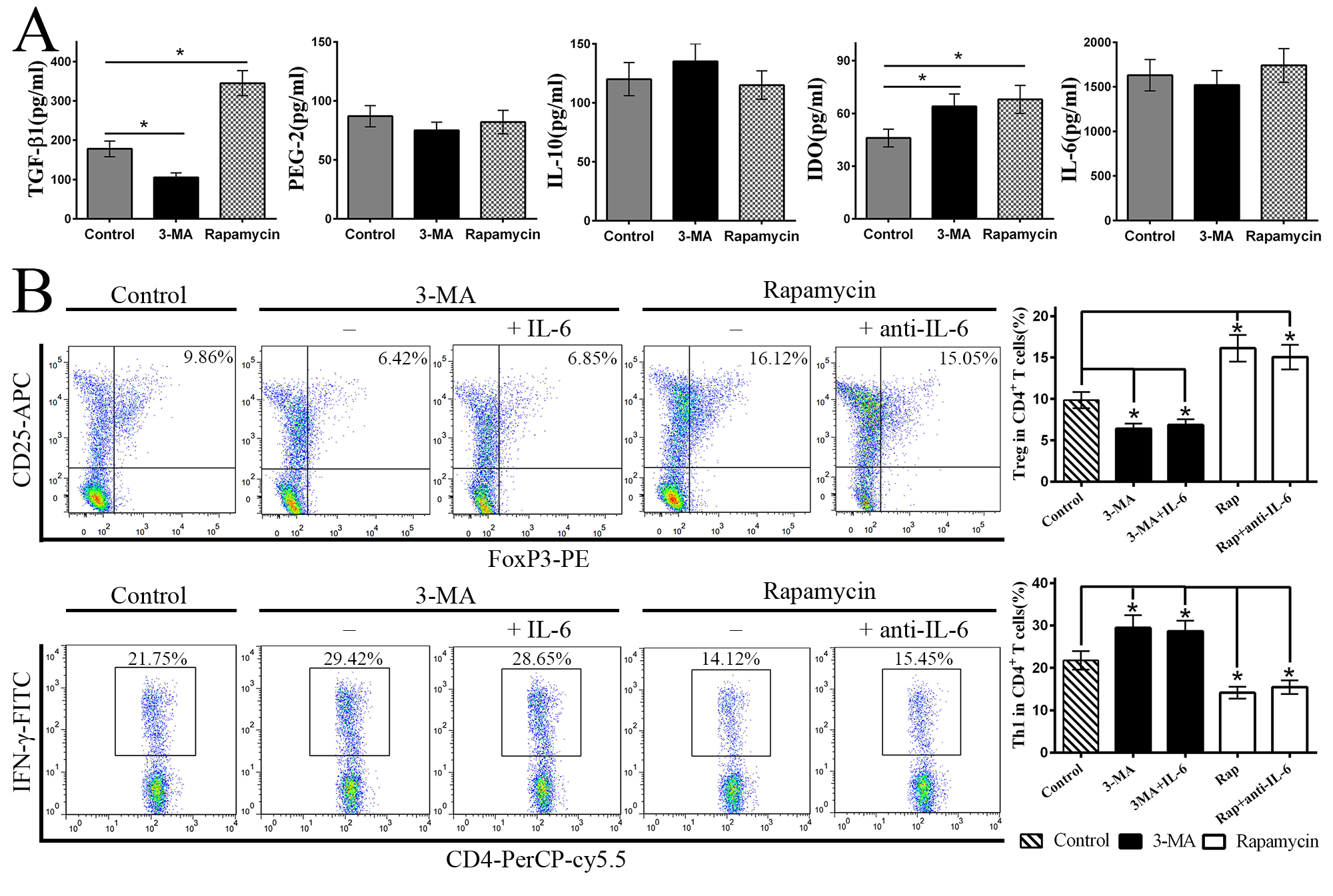

Supplement: Supplementary file 4 — Figure S4. The secretion patterns of the immunoregulatory factors in MSCs are affected by autophagy. (A) The secretion levels of TGF-β1, PEG-2, IL-10, IDO and IL-6 in MSCs were measured by using Elisa. Only TGF-β1 secretion showed a positive correlation with MSC autophagy. (B) Exogenous IL-6 protein and antibody were added to the 3-MA and rapamycin group respectively to assessed its role in the MSC-mediated CD4+ T cell polarization. The flow cytometry results revealed that exogenous IL-6 protein or anti-IL-6 antibody exerted little effect on the MSC-mediated Treg and Th1 cell polarization. Values are presented as the means ± SD of 18 samples per group. * indicates P < 0.05. (TIF 9965 kb) [file 13287_2019_1380_MOESM4_ESM.tif]

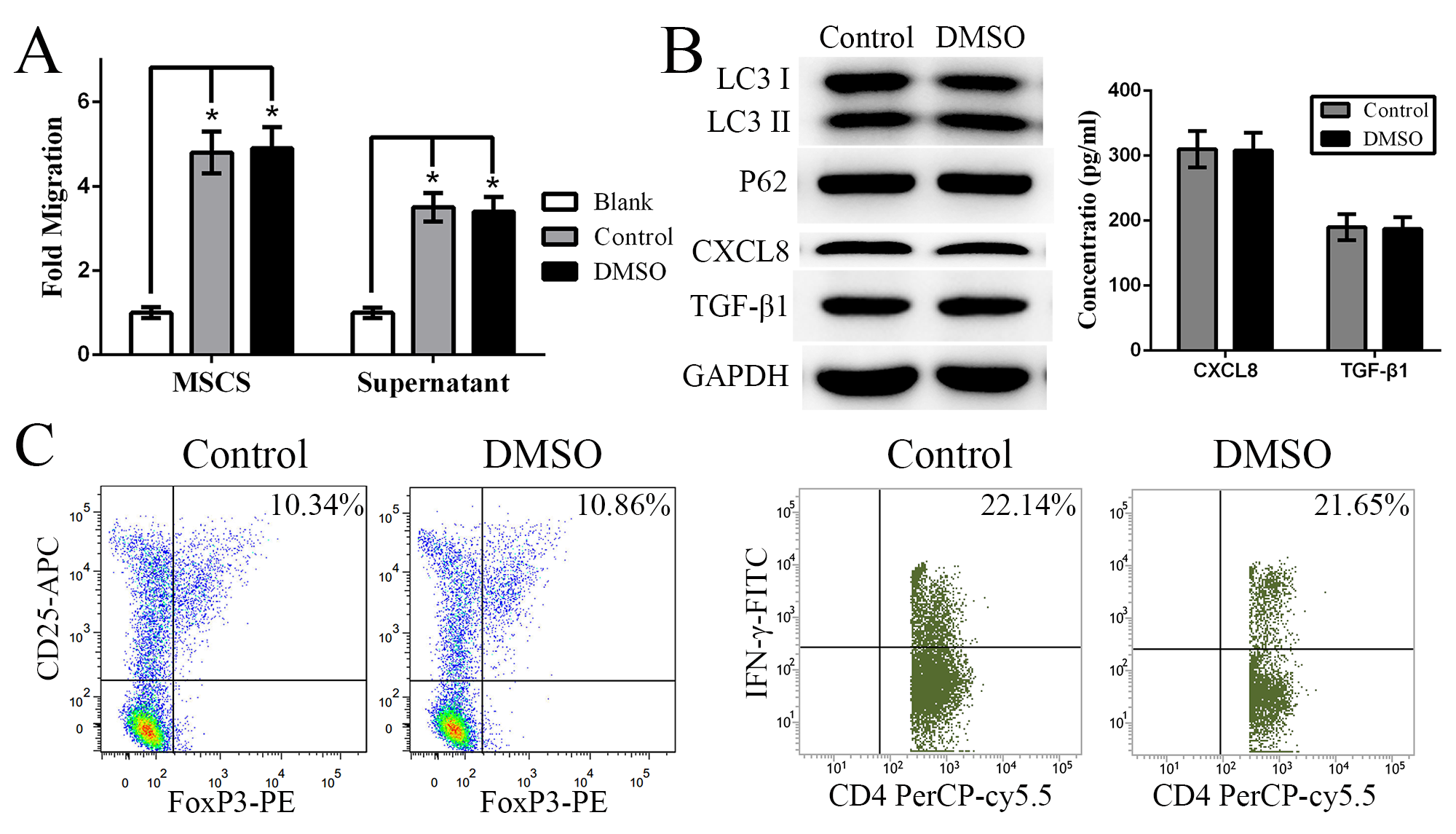

Supplement: Supplementary file 5 — Figure S5. DMSO does not exert visible effects on MSCs compared with the control group. (A) The number of migrated CD4+ T cells was analyzed by flow cytometry. DMSO did not affect the MSC-mediated CD4+ T cell migration, and the DMSO-pretreated MSC culture supernatants showed similar CD4+ T cell migration compared with the control group. (B) The expression and secretion of CXCL8 and TGF-β1 were similar between the DMSO-pretreated and control group. There was no difference in autophagy between DMSO-pretreated and control group, detected by western blotting targeting LC3 and P62. (C) The flow cytometry showed that DMSO did not affect the MSC-mediated Treg and Th1 polarization. Values are presented as the means ± SD of 18 samples per group. * indicates P < 0.05. (TIF 8346 kb) [file 13287_2019_1380_MOESM5_ESM.tif]
