# Supplementary material for: Autophagy enhances mesenchymal stem cell-mediated CD4+ T cell migration and differentiation through CXCL8 and TGF-β1
Source: Stem Cell Res Ther. 2019 Aug 23;10:265. doi: 10.1186/s13287-019-1380-0 (PMC6708254; doi:10.1186/s13287-019-1380-0)
Supplement: Supplementary file 6 — Table S1. Primers used for qRT-PCR (DOCX 16 kb) [file 13287_2019_1380_MOESM6_ESM.docx]

| Gene Accession No. Forward primer Reverse primer Product  (5’-3’) (5’-3’) size(bp) |
| --- |
| GAPDH NM_001256799 GGAGCGAGATCCCTCCAAAAT GGCTGTTGTCATACTTCTCATGG 197  TGF-β1 NM_000660 CAATTCCTGGCGATACCTCAG GCACAACTCCGGTGACATCAA 201  CXCL8 [NM_000584.3](https://www.ncbi.nlm.nih.gov/nuccore/NM_000584.3)  ACTGAGAGTGATTGAGAGTGGAC AACCCTCTGCACCCAGTTTTC 112  CXCL16 [NM_001100812.1](https://www.ncbi.nlm.nih.gov/nuccore/NM_001100812.1) CCCGCCATCGGTTCAGTTC CCCCGAGTAAGCATGTCCAC 181  CXCL5 [NM_002994.4](https://www.ncbi.nlm.nih.gov/entrez/viewer.fcgi?db=nucleotide&id=493799665) AGCTGCGTTGCGTTTGTTTAC TGGCGAACACTTGCAGATTAC 75  CXCL1 [NM_001511.3](https://www.ncbi.nlm.nih.gov/nuccore/NM_001511.3) AACATGCCAGCCACTGTGAT GCCCCTTTGTTCTAAGCCAG 287  CXCL10 [NM_001565.3](https://www.ncbi.nlm.nih.gov/entrez/viewer.fcgi?db=nucleotide&id=323422857) GTGGCATTCAAGGAGTACCTC TGATGGCCTTCGATTCTGGATT 198  CXCL2 [NM_002089.3](https://www.ncbi.nlm.nih.gov/entrez/viewer.fcgi?db=nucleotide&id=148298657) TGTGACGGCAGGGAAATGTA TCTGCTCTAACACAGAGGGAAAC 225  CCL19 [NM_006274.2](https://www.ncbi.nlm.nih.gov/entrez/viewer.fcgi?db=nucleotide&id=22165424) TACATCGTGAGGAACTTCCACT CTGGATGATGCGTTCTACCCA 132  CXCL4 [NM_002619.3](https://www.ncbi.nlm.nih.gov/entrez/viewer.fcgi?db=nucleotide&id=380254445) ACTAGCTGCCTACGTGTGTG GCATAACCAGTATTCACACCTTCC 259  CCL5 [NM_001278736.1](https://www.ncbi.nlm.nih.gov/entrez/viewer.fcgi?db=nucleotide&id=524563422) CAGTCGTCCACAGGTCAAGG TCTTCTCTGGGTTGGCACAC 136  CXCL12 [NM_001178134](http://www.ncbi.nlm.nih.gov/entrez/query.fcgi?cmd=Search&db=Nucleotide&term=NM_001178134) ATTCTCAACACTCCAAACTGTGC ACTTTAGCTTCGGGTCAATGC 88 |

**Supplemental Table 1** Primers used for qRT-PCR
